# Large Language Models for Mental Health Applications: Systematic Review

**DOI:** 10.2196/57400

**Published:** 2024-10-18

**Authors:** Zhijun Guo, Alvina Lai, Johan H Thygesen, Joseph Farrington, Thomas Keen, Kezhi Li

**Affiliations:** 1 Institute of Health Informatics University College, London London United Kingdom; 2 Great Ormond Street Institute of Child Health University College London London United Kingdom

**Keywords:** large language models, mental health, digital health care, ChatGPT, Bidirectional Encoder Representations from Transformers, BERT

## Abstract

**Background:**

Large language models (LLMs) are advanced artificial neural networks trained on extensive datasets to accurately understand and generate natural language. While they have received much attention and demonstrated potential in digital health, their application in mental health, particularly in clinical settings, has generated considerable debate.

**Objective:**

This systematic review aims to critically assess the use of LLMs in mental health, specifically focusing on their applicability and efficacy in early screening, digital interventions, and clinical settings. By systematically collating and assessing the evidence from current studies, our work analyzes models, methodologies, data sources, and outcomes, thereby highlighting the potential of LLMs in mental health, the challenges they present, and the prospects for their clinical use.

**Methods:**

Adhering to the PRISMA (Preferred Reporting Items for Systematic Reviews and Meta-Analyses) guidelines, this review searched 5 open-access databases: MEDLINE (accessed by PubMed), IEEE Xplore, Scopus, JMIR, and ACM Digital Library. Keywords used were (*mental health* OR *mental illness* OR *mental disorder* OR *psychiatry*) AND (*large language models*). This study included articles published between January 1, 2017, and April 30, 2024, and excluded articles published in languages other than English.

**Results:**

In total, 40 articles were evaluated, including 15 (38%) articles on mental health conditions and suicidal ideation detection through text analysis, 7 (18%) on the use of LLMs as mental health conversational agents, and 18 (45%) on other applications and evaluations of LLMs in mental health. LLMs show good effectiveness in detecting mental health issues and providing accessible, destigmatized eHealth services. However, assessments also indicate that the current risks associated with clinical use might surpass their benefits. These risks include inconsistencies in generated text; the production of hallucinations; and the absence of a comprehensive, benchmarked ethical framework.

**Conclusions:**

This systematic review examines the clinical applications of LLMs in mental health, highlighting their potential and inherent risks. The study identifies several issues: the lack of multilingual datasets annotated by experts, concerns regarding the accuracy and reliability of generated content, challenges in interpretability due to the “black box” nature of LLMs, and ongoing ethical dilemmas. These ethical concerns include the absence of a clear, benchmarked ethical framework; data privacy issues; and the potential for overreliance on LLMs by both physicians and patients, which could compromise traditional medical practices. As a result, LLMs should not be considered substitutes for professional mental health services. However, the rapid development of LLMs underscores their potential as valuable clinical aids, emphasizing the need for continued research and development in this area.

**Trial Registration:**

PROSPERO CRD42024508617; https://www.crd.york.ac.uk/prospero/display_record.php?RecordID=508617

## Introduction

### Mental Health

Mental health, a critical component of overall well-being, is at the forefront of global health challenges [1]. In 2019, an estimated 970 million individuals worldwide experienced mental illness, accounting for 12.5% of the global population [2]. Anxiety and depression are among the most prevalent psychological conditions, affecting 301 million and 280 million individuals, respectively [2]. In addition, 40 million people experienced bipolar disorder, 24 million experienced schizophrenia, and 14 million experienced eating disorders [3]. These mental disorders collectively contribute to an estimated US $5 trillion in global economic losses annually [4]. Despite the staggering prevalence, many cases remain undetected or untreated, with the resources allocated to the diagnosis and treatment of mental illness far less than the negative impact it has on society [5]. Globally, untreated mental illnesses affect 5% of the population in high-income countries and 19% of the population in low- and middle-income countries [3]. The COVID-19 pandemic has further exacerbated the challenges faced by mental health services worldwide [6], as the demand for these services increased while access was decreased [7]. This escalating crisis underscores the urgent need for more innovative and accessible mental health care approaches.

Mental illness treatment encompasses a range of modalities, including medication, psychotherapy, support groups, hospitalization, and complementary and alternative medicine [8]. However, the societal stigma attached to mental illnesses often deters people from seeking appropriate care [9]. Influenced by the fear of judgment and concerns about costly, ineffective treatments [10], many people with mental illness avoid or delay psychotherapy [11]. The COVID-19 crisis and other global pandemics have underscored the importance of digital tools, such as telemedicine and mobile apps, in delivering care during critical times [12]. In this evolving context, large language models (LLMs) present new possibilities for enhancing the delivery and effectiveness of mental health care.

Recent technological advancements have revealed some unique advantages of LLMs in mental health. These models, capable of processing and generating text akin to human communication, provide accessible support directly to users [13]. A study analyzing 2917 Reddit (Reddit, Inc) user reviews found that conversational agents (CAs) powered by LLMs are valued for their nonjudgmental listening and effective problem-solving advice. This aspect is particularly beneficial for individuals considered socially marginalized, as it enables them to be heard and understood without the need for direct social interaction [14]. Moreover, LLMs enhance the accessibility of mental health services, which are notably undersupplied globally [15]. Recent data reveals substantial delays in traditional mental health care delivery; 23% of individuals with mental illnesses report waiting for >12 weeks for face-to-face psychotherapy sessions [16], with 12% waiting for >6 months and 6% waiting for >1 year [16]. In addition, 43% of adults with mental illness indicate that such long waits have exacerbated their conditions [16].

Telemedicine, enhanced by LLMs, offers a practical alternative that expedites service delivery and could flatten traditional health care hierarchies [17]. This includes real-time counseling sessions through CAs that are not only cost-effective but also accessible anytime and from any location. By reducing the reliance on physical visits to traditional health care settings, telemedicine has the potential to decentralize access to medical expertise and diminish the hierarchical structures within the health care system [17]. Mental health chatbots developed using language models, such as Woebot [18] and Wysa [19], have been gaining recognition. Both chatbots follow the principles of cognitive behavioral therapy and are designed to equip users with self-help tools for managing their mental health issues [20]. In clinical practice, LLMs hold the potential to support the automatic assessment of therapists’ adherence to evidence-based practices and the development of systems that offer real-time feedback and support for patient homework between sessions [21]. These models also have the potential to provide feedback on psychotherapy or peer support sessions, which is especially beneficial for clinicians with less training and experience [21]. Currently, these applications are still in the proposal stage. Although promising, they are not yet widely used in routine clinical settings, and further evaluation of their feasibility and effectiveness is necessary.

The deployment of LLMs in mental health also poses several risks, particularly for groups considered vulnerable. Challenges such as inconsistencies in the content generated and the production of “hallucinatory” content may mislead or harm users [22], raising serious ethical concerns. In response, authorities such as the World Health Organization have developed ethical guidelines for artificial intelligence (AI) research in health care, emphasizing the importance of data privacy; human oversight; and the principle that AI tools should augment, rather than replace, human practitioners [23]. These potential problems with LLMs in health care have gained considerable industry attention, underscoring the need for a comprehensive and responsible evaluation of LLMs’ applications in mental health. The following section will further explore the workings of LLMs and their potential applications in mental health and critically evaluate the opportunities and challenges they introduce.

### LLMs in Mental Health

LLMs represent advancements in machine learning, characterized by their ability to understand and generate human-like text with high accuracy [24]. The efficacy of these models is typically evaluated using benchmarks designed to assess their linguistic fidelity and contextual relevance. Common metrics include Bilingual Evaluation Understudy for translation accuracy and Recall-Oriented Understudy for Gisting Evaluation (ROUGE) for summarization tasks [25]. LLMs are characterized by their scale, often encompassing billions of parameters, setting them apart from traditional language models [26]. This breakthrough is largely due to the transformer architecture, a deep neural network structure that uses a “self-attention” mechanism developed by Vaswani et al [27]. This allows LLMs to process information in parallel rather than sequentially, greatly enhancing speed and contextual understanding [27]. To clearly define the scope of this study concerning LLMs, we specify that an LLM must use the transformer architecture and contain a high number of parameters, traditionally at least 1 billion, to qualify as “large” [28]. This criterion encompasses models such as GPT (OpenAI) and Bidirectional Encoder Representations from Transformers (BERT; Google AI). Although the standard BERT model, with only 0.34 billion parameters [29], does not meet the traditional criteria for “large,” its sophisticated bidirectional design and pivotal role in establishing new natural language processing (NLP) benchmarks justify its inclusion among notable LLMs [30]. The introduction of ChatGPT (OpenAI) in 2022 generated substantial public and academic interest in LLMs, underlining their transformative potential within the field of AI [31]. Other state-of-the-art LLMs include Large Language Model Meta AI (LLaMA; Meta AI) and Pathways Language Model (PaLM; Google AI), as illustrated in Table 1 [32-35].

**Table 1:** Comparative analysis of large language models (LLMs) by parameter size and developer entity. Data were summarized with the latest models up to June 2024, with data for parameters and developers from GPT (OpenAI) to Large Language Model Meta AI (LLaMA; Meta AI) adapted from the study by Thirunavukarasu et al [32].

Model name	Publication date	Parameters (billion)	Developer
Generative Pretrained Transformer (GPT)	June 2018	0.117	OpenAI
Bidirectional Encoder Representations from Transformers (BERT)	October 2018	0.34	Google
GPT-2	January 2019	1.5	OpenAI
Enhanced Representation through Knowledge Integration (ERNIE)	September 2019	0.114	Baidu
Conditional Transformer Language Model (CTRL)	September 2019	1.63	OpenAI
Megatron	September 2019	3.9	NVIDIA
Bidirectional and Auto-Regressive Transformers (BART)	October 2019	0.374	Meta
Turing Natural Language Generation (Turing-NLG)	January 2020	530	Microsoft
GPT-3	June 2020	175	OpenAI
Vision Transformer (ViT)	October 2020	0.632	Google
Inspired by artist Salvador Dalí and Pixar's WALL·E (DALL-E)	October 2020	1.2	OpenAI
Swin Transformer	March 2021	0.197	Microsoft
Wu Dao 2.0	June 2021	1750	Huawei
Jurassic-1	August 2021	178	AI21 Labs
Megatron-Turing Natural Language Generation (MT-NLG)	October 2021	530	Microsoft & Nvidia
Claude	December 2021	52	Anthropic
Generalist Language Model (GLAM)	December 2021	1200	Google
ERNIE 3.0	December 2021	260	Baidu
Guided Language-to-Image Diffusion for Generation and Editing (GLIDE)	December 2021	3.5	OpenAI
Gopher	December 2021	280	DeepMind
Causal Masked Modeling 3 (CM3)	January 2022	13	Meta
Language Model for Dialogue Applications (LaMDA)	January 2022	137	Google
GPT-NeoX	February 2022	20	EleutherAI
Chinchilla	March 2022	70	DeepMind
GopherCite	March 2022	280	DeepMind
DALL-E 2	April 2022	3.5	OpenAI
Flamingo	April 2022	80	DeepMind
Pathways Language Model (PaLM)	April 2022	540	Google
Gato	May 2022	1.2	DeepMind
Open Pretrained Transformer (OPT)	May 2022	175	Meta
Yet Another Language Model (YaLM)	June 2022	100	Yandex
Minerva	June 2022	540	Google
BigScience Large Open-science Open-access Multilingual Language Model (BLOOM)	July 2022	175	Hugging Face
Galactica	November 2022	120	Meta
Alexa Teacher Model (Alexa TM)	November 2022	20	Amazon
Large Language Model Meta AI (LLAMA)	February 2023	65	Meta
GPT-4	March 2023	1760	OpenAI
Cerebras-GPT	March 2023	13	Cerebras
Falcon	March 2023	40	Technology Innovation Institute
Bloomberg Generative Pretrained Transformer (BloombergGPT)	March 2023	50	Bloomberg
PanGu-2	March 2023	1085	Huawei
OpenAssistant	March 2023	17	LAION
PaLM 2	May 2023	340	Google
Llama 2	July 2023	70	Meta
Falcon 180B	September 2023	180	Technology Innovation Institute
Mistral 7B	September 2023	7.3	Mistral
Claude 2.1	November 2023	200	Anthropic
Grok-1	November 2023	314	xAI
Mixtral 8x7B	December 2023	46.7	Mistral
Phi-2	December 2023	2.7	EleutherAI
Gemma	February 2024	7	Google
DBRX	March 2024	136	Databricks
Llama 3	April 2024	70	Meta AI
Fugaku-LLM	May 2024	13	Fujitsu, Tokyo Institute of Technology, etc
Nemotron-4	June 2024	340	Nvidia

LLMs are primarily designed to learn fundamental statistical patterns of language [36]. Initially, these models were used as the basis for fine-tuning task-specific models rather than training those models from scratch, offering a more resource-efficient approach [37]. This fine-tuning process involves adjusting a pretrained model to a specific task by further training it on a smaller, task-specific dataset [38]. However, developments in larger and more sophisticated models have reduced the need for extensive fine-tuning in some cases. Notably, some advanced LLMs can now effectively understand and execute tasks specified through natural language prompts without extensive task-specific fine-tuning [39]. Instruction fine-tuned models undergo additional training on pairs of user requests and appropriate responses. This training allows them to generalize across various complex tasks, such as sentiment analysis, which previously required explicit fine-tuning by researchers or developers [40]. A key part of the input to these models, such as ChatGPT and Gemini (Google AI), includes a system prompt, often hidden from the user, which guides the model on how to interpret and respond to user prompts. For example, it might direct the model to act as a helpful mental health assistant. In addition, “prompt engineering” has emerged as a crucial technique in optimizing model performance. Prompt engineering involves crafting input texts that guide the model to produce the desired output without additional training. For example, refining a prompt from “Tell me about current events in health care” to “Summarize today’s top news stories about technology in health care” provides the model with more specific guidance, which can enhance the relevance and accuracy of its responses [41]. While prompt engineering can be highly effective and reduce the need to retrain the model, it is important to be wary of “hallucinations,” a phenomenon where models confidently generate incorrect or irrelevant outputs [42]. This can be particularly challenging in high-accuracy scenarios, such as health care and medical applications [43-46]. Thus, while prompt engineering reduces the reliance on extensive fine-tuning, it underscores the need for thorough evaluation and testing to ensure the reliability of model outputs in sensitive applications.

The existing literature includes a review of the application of machine learning and NLP in mental health [47], analyses of LLMs in medicine [32], and a scoping review of LLMs in mental health. These studies have demonstrated the effectiveness of NLP for tasks such as text categorization and sentiment analysis [47] and provided a broad overview of LLM applications in mental health [48]. However, a gap remains in systematically reviewing state-of-the-art LLMs in mental health, particularly in the comprehensive assessment of literature published since the introduction of the transformer architecture in 2017.

This systematic review addresses these gaps by providing a more in-depth analysis; evaluating the quality and applicability of studies; and exploring ethical challenges specific to LLMs, such as data privacy, interpretability, and clinical integration. Unlike previous reviews, this study excludes preprints, follows a rigorous search strategy with clear inclusion and exclusion criteria (using Cohen κ to assess the interreviewer agreement), and uses a detailed assessment of study quality and bias (using the Risk of Bias 2 tool) to ensure the reliability and reproducibility of the findings.

Guided by specific research questions, this systematic review critically assesses the use of LLMs in mental health, focusing on their applicability and efficacy in early screening, digital interventions, and clinical settings, as well as the methodologies and data sources used. The findings of this study highlight the potential of LLMs in enhancing mental health diagnostics and interventions while also identifying key challenges such as inconsistencies in model outputs and the lack of robust ethical guidelines. These insights suggest that, while LLMs hold promise, their use should be supervised by physicians, and they are not yet ready for widespread clinical implementation.

## Methods

This systematic review followed the PRISMA (Preferred Reporting Items for Systematic Review and Meta-Analyses) guidelines [49]. The protocol was registered on PROSPERO (CRD42024508617). A PRISMA checklist is available in Multimedia Appendix 1.

### Search Strategies

The search was initiated on August 3, 2024, and completed on August 6, 2024, by 1 author (ZG). ZG systematically searched 5 databases: MEDLINE, IEEE Xplore, Scopus, JMIR, and ACM Digital Library using the following search keywords: (*mental health* OR *mental illness* OR *mental disorder* OR *psychiatry*) and (*large language models*). These keywords were consistently applied across each database to ensure a uniform search strategy. To conduct a comprehensive and precise search for relevant literature, strategies were tailored for different databases. All metadata were searched in MEDLINE and IEEE Xplore, whereas the search in Scopus was confined to titles, abstracts, and keywords. The JMIR database used the criteria *exact match* feature to refine search results and enhance precision. In the ACM Digital Library database, the search focused on full text. The screening of all citations involved four steps:

1. Initial search. All relevant citations were imported into a Zotero (Corporation for Digital Scholarship) citation manager library.
2. Preliminary inclusion. Citations were initially screened based on predefined inclusion criteria.
3. Duplicate removal. Citations were consolidated into a single group, from which duplicates were eliminated.
4. Final inclusion. The remaining references were carefully evaluated against the inclusion criteria to determine their suitability.

### Study Selection and Eligibility Criteria

All the articles that matched the search criteria were double screened by 2 independent reviewers (ZG and KL) to ensure that each article fell within the scope of LLMs in mental health. This process involved the removal of duplicates followed by a detailed manual evaluation of each article to confirm adherence to our predefined inclusion criteria, ensuring a comprehensive and focused review. To quantify the agreement level between the reviewers and ensure objectivity, interrater reliability was calculated using Cohen κ, with a score of 0.84 indicating a good level of agreement. In instances of disagreement, a third reviewer (AL) was consulted to achieve consensus.

To assess the risk of bias, we used the Risk of Bias 2 tool, as recommended for Cochrane Reviews. The results have been visualized in Multimedia Appendix 2. We thoroughly examined each study for potential biases that could impact the validity of the results. These included biases from the randomization process, deviations from intended interventions, missing outcome data, inaccuracies in outcome measurement, and selective reporting of results. This comprehensive assessment ensures the credibility of each study.

The criteria for selecting articles were as follows: we limited our search to English-language publications, focusing on articles published between January 1, 2017, and April 30, 2024. This timeframe was chosen considering the substantial developments in the field of LLMs in 2017, marked notably by the introduction of the transformer architecture, which has greatly influenced academic and public interest in this area.

In this review, the original research articles and available full-text papers have been carefully selected, aiming to focus on the application of LLMs in mental health. To comply with the PRISMA guidelines, articles that have not been published in a peer-reviewed venue, including those only available on a preprint server, were excluded. Owing to the limited literature specifically addressing the mental health applications of LLMs, we included review articles to ensure a comprehensive perspective. The selection criteria focused on direct applications, expert evaluations, and ethical considerations related to the use of LLMs in mental health contexts, with the goal of providing a thorough analysis of this rapidly developing field.

### Information Extraction

The data extraction process was jointly conducted by 2 reviewers (ZG and KL), focusing on examining the application scenarios, model architecture, data sources, methodologies used, and main outcomes from selected studies on LLMs in mental health.

Initially, we categorized each study to determine its main objectives and applications. The categorization process was conducted in 2 steps. First, after reviewing all the included articles, we grouped them into 3 primary categories: detection of mental health conditions and suicidal ideation through text, LLM use for mental health CAs, and other applications and evaluation of the LLMs in mental health. In the second step, we performed a more detailed categorization. After a thorough, in-depth reading of each article within these broad categories, we refined the classifications based on the specific goals of the studies. Following this, we summarized the main model architectures of the LLMs used and conducted a thorough examination of data sources, covering both public and private datasets. We noted that some review articles lacked detail on dataset content; therefore, we focused on providing comprehensive information on public datasets, including their origins and sample sizes. We also investigated the various methods used across different studies, including data collection strategies and analytic methodologies. We examined their comparative structures and statistical techniques to offer a clear understanding of how these methods are applied in practice.

Finally, we documented the main outcomes of each study, recording significant results and aligning them with relevant performance metrics and evaluation criteria. This included providing quantitative data where applicable to underscore these findings. We used a narrative approach to synthesize the information, integrating and comparing results from various studies to emphasize the efficacy and impact of LLMs on mental health. This narrative synthesis allowed us to highlight the efficacy and impact of LLMs in mental health, providing quantitative data where applicable to underscore these findings. The results of this analysis are presented in Tables S1-S3 in Multimedia Appendix 3 [14,50-131], each corresponding to 1 of the primary categories.

## Results

### Strategy and Screening Process

**Figure 1:**
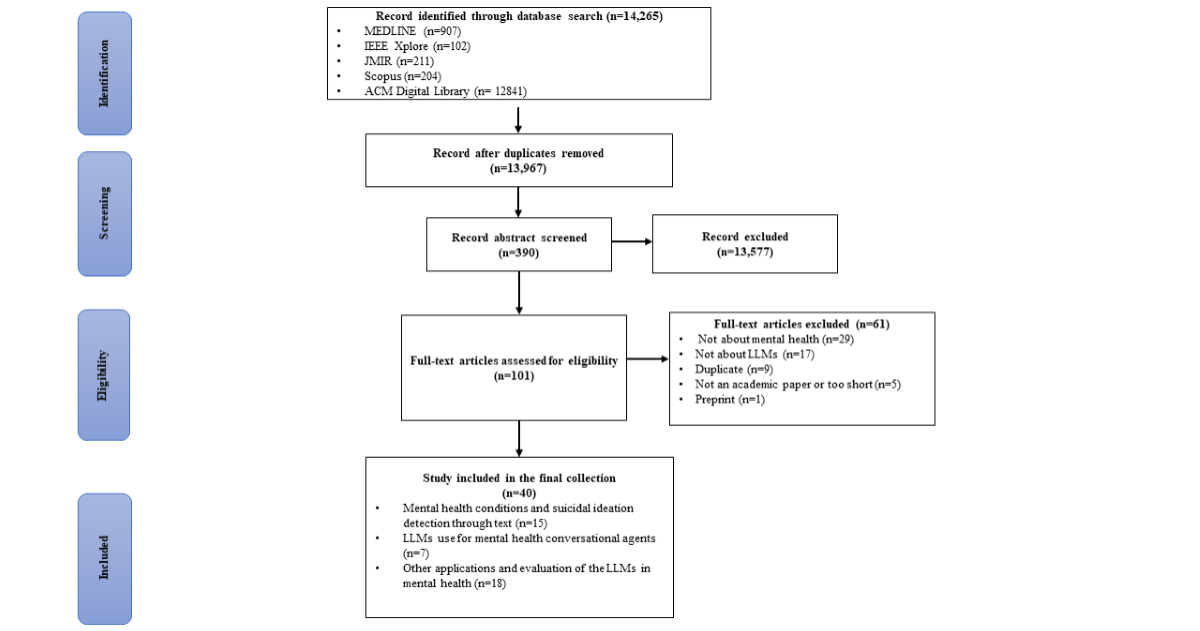
PRISMA (Preferred Reporting Items for Systematic Reviews and Meta-Analyses) flow of the selection process. LLM: large language model.

The PRISMA diagram of the systematic screening process can be seen in Figure 1. Our initial search across 5 academic databases, namely, MEDLINE, IEEE Xplore, Scopus, JMIR, and ACM Digital Library, yielded 14,265 papers: 907 (6.36%) from MEDLINE, 102 (0.72%) from IEEE Xplore, 204 (1.43%) from Scopus, 211 (1.48%) from JMIR, and 12,841 (90.02%) from ACM Digital Library. After duplication, 97.91% (13,967/14,265) of the unique papers were retained. Subsequent screening was based on predefined inclusion and exclusion criteria, narrowing down the selection to 0.29% (40/13,967) of the papers included in this review. The reasons for the full-text exclusion of 61 papers can be found in Multimedia Appendix 4.

In our review of the literature, we classified the included articles into 3 broad categories: detection of mental health conditions and suicidal ideation through text (15/40, 38%), LLMs’ use for mental health CAs (7/40, 18%), and the other applications and evaluation of the LLMs in mental health (18/40, 45%). The first category investigates the potential of LLMs for the early detection of mental illness and suicidal ideation via social media and other textual sources. Early screening is highlighted as essential for preventing the progression of mental disorders and mitigating more severe outcomes. The second category assesses LLM-supported CAs used as teletherapeutic interventions for mental health issues, such as loneliness, with a focus on evaluating their effectiveness and validity. The third category covers a broader range of LLM applications in mental health, including clinical uses such as decision support and therapy enhancement. It aims to assess the overall effectiveness, utility, and ethical considerations associated with LLMs in these settings. All selected articles are summarized in Tables S1-S3 in Multimedia Appendix 3 according to the 3 categories.

### Mental Health Conditions and Suicidal Ideation Detection Through Text

Early intervention and screening are crucial in mitigating the global burden of mental health issues [132]. We examined the performance of LLMs in detecting mental health conditions and suicidal ideation through textual analysis. Of 40 articles, 6 (15%) assessed the efficacy of early screening for depression using LLMs [50,57,60,61,66,68], while another (1/40, 2%) simultaneously addressed both depression and anxiety [60]. One comprehensive study examined various psychiatric conditions, including depression, social anxiety, loneliness, anxiety, and other prevalent mental health issues [69]. Two (5%) of the 40 articles assessed and compared the ability of LLMs to perform sentiment and emotion analysis [75,81], and 5 (12%) articles focused on the capability of LLMs to analyze textual content for detecting suicidal ideation [54,65,70,72,78]. Most studies (10/40, 25%) used BERT and its variants as one of the primary models [50,54,57,62,65,66,68,69,75,78], while GPT models were also commonly used (8/40, 20%) [57,60,61,66,70,72,78,81]. Most training data (10/40, 25%) comprised social media posts [50,54,62,65,68,69,72,75,78,81] from platforms such as Twitter (Twitter, Inc), Reddit, and Sina Weibo (Sina corporation), covering languages such as English, Malay dialects, Chinese, and Portuguese. In addition, 5 (12%) of the 40 studies used datasets consisting of clinical transcripts and patient interviews [50,57,60,61,66], providing deeper insights into LLM applications in clinical mental health settings.

In studies focusing on early screening for depression, comparing results horizontally is challenging due to variations in datasets, training methods, and models across different investigations. Nonetheless, substantial evidence supports the significant potential of LLMs in detecting depression from text-based data. For example, Danner et al [57] conducted a comparative analysis using a convolutional neural network on the Distress Analysis Interview Corpus-Wizard of Oz dataset, achieving *F*_1_-scores of 0.53 and 0.59; however, their use of GPT-3.5 demonstrated superior performance, with an *F*_1_-score of 0.78. Another study involving the E-Distress Analysis Interview Corpus dataset (an extension of Distress Analysis Interview Corpus-Wizard of Oz) used the Robustly Optimized BERT Approach for Depression Detection to predict the Patient Health Questionnaire-8 scores from textual data. This approach identified 3 levels of depression and achieved the lowest mean absolute error of 3.65 in Patient Health Questionnaire–8 scores [66].

LLMs play an important role in sentiment analysis [75,81], which categorizes text into overall polarity classes, such as positive, neutral, negative, and occasionally mixed, and emotion classification, which assigns labels such as “joy,” “sadness,” “anger,” and “fear” [75]. These analyses enable the detection of emotional states and potential mental health issues from textual data, facilitating early intervention [133]. Stigall et al [75] demonstrated the efficacy of these models, with their study showing that Emotion-aware BERT Tiny, a fine-tuned variant of BERT, achieved an accuracy of 93.14% in sentiment analysis and 85.46% in emotion analysis. This performance surpasses that of baseline models, including BERT-Base Cased and BERTTiny-Pretrained [75], underscoring the advantages and validity of fine-tuning in enhancing model performance. LLMs have also demonstrated robust accuracy in detecting and classifying a range of mental health syndromes, including social anxiety, loneliness, and generalized anxiety. Vajre et al [69] introduced PsychBERT, developed using a diverse training dataset from both social media texts and academic literature, which achieved an *F*_1_-score of 0.63, outperforming traditional deep learning approaches such as convolutional neural networks and long short-term memory networks, which recorded *F*_1_-scores of 0.57 and 0.51, respectively [69]. In research on detecting suicidal ideation using LLMs, Diniz et al [54] showcased the high efficacy of the BERTimbau large model within a non-English (Portuguese) context, achieving an accuracy of 0.955, precision of 0.961, and an *F*_1_-score of 0.954. The assessment of the BERT model by Metzler et al [65] found that it correctly identified 88.5% of tweets as suicidal or off-topic, performing comparably to human analysts and other leading models. However, Levkovich et al [70] noted that while GPT-4 assessments of suicide risk closely aligned with those by mental health professionals, it overestimated suicidal ideation. These results underscore that while LLMs have the potential to identify tweets reflecting suicidal ideation with accuracy comparable to psychological professionals, extensive follow-up studies are required to establish their practical application in clinical settings.

### LLMs in Mental Health CAs

In the growing field of mental health digital support, the implementation of LLMs as CAs has exhibited both promising advantages [14,84,91,96] and significant challenges [92,96]. The studies by Ma et al [14] and Heston [96] demonstrate the effectiveness of CAs powered by LLMs in providing timely, nonjudgmental mental health support. This intervention is particularly important for those who lack ready access to a therapist due to constraints such as time, distance, and work, as well as for certain populations considered socially marginalized, such as older adults who experience chronic loneliness and a lack of companionship [14,97]. The qualitative analysis of user interactions on Reddit by Ma et al [14] highlights that LLMs encourage users to speak up and boost their confidence by providing personalized and responsive interactions. In addition, VHope, a DialoGPT-enabled mental health CA, was evaluated by 3 experts who rated its responses as 67% relevant, 78% human-like, and 79% empathetic [84]. Another study found that after observing 717 evaluations by 100 participants on 239 autism-specific questions, 46.86% of evaluators preferred responses of the chief physicians, whereas 34.87% preferred the responses of GPT-4, and 18.27% favored the responses of Enhanced Representation through Knowledge Integration Bot (ERNIE Bot; version 2.2.3; Baidu, Inc). Moreover, ChatGPT (mean 3.64, 95% CI 3.57-3.71) outperformed physicians (mean 3.13, 95% CI 3.04-3.21) in terms of empathy [98], indicating that LLM-powered CAs are not only effective but also acceptable by users. These findings highlight the potential for LLMs to complement mental health intervention systems and provide valuable medical guidance.

The development and implementation of a non-English CA for emotion capture and categorization was explored in a study by Zygadlo et al [92]. Faced with a scarcity of Polish datasets, the study adapted by translating an existing database of personal conversations from English into Polish, which decreased the accuracy in tasks from 90% in English to 80% in Polish [92]. While the performance remained commendable, it highlighted the challenges posed by the lack of robust datasets in languages other than English, impacting the effectiveness of CAs across different linguistic environments. However, findings by He et al [98] suggest that the availability of language-specific datasets is not the sole determinant of CA performance. In their study, although ERNIE Bot was trained in Chinese and ChatGPT in English, ChatGPT demonstrated greater empathy for Chinese users [98]. This implies that factors beyond the training language and dataset availability, such as model architecture or training methodology, can also affect the empathetic responsiveness of LLMs, underscoring the complexity of human-AI interaction.

Meanwhile, the reliability of LLM-driven CAs in high-risk scenarios remains a concern [14,96]. An evaluation of 25 CAs found that in tests involving suicide scenarios, only 2 included suicide hotline referrals during the conversation [96]. This suggests that while these CAs can detect extreme emotions, few are equipped to take effective preventive measures. Furthermore, CAs often struggle with maintaining consistent communication due to limited memory capacity, leading to disruptions in conversation flow and negatively affecting user experience [14].

### Other Applications and Evaluation of the LLMs in Mental Health

ChatGPT has gained attention for its unparalleled ability to generate human-like text and analyze large amounts of textual data, attracting the interest of many researchers and practitioners [100]. Numerous evaluations of LLMs in mental health have focused on ChatGPT, exploring its utility across various scenarios such as clinical diagnosis [100,106,111], treatment planning [106,128,131], medication guidance [105,109,129], patient management [106], psychiatry examinations [118], and psychology education [102], among others [107,110,127,130].

Research has highlighted ChatGPT’s accuracy in diagnosing various psychiatric conditions [106,110,111,126]. For example, Franco D’Souza et al [100] evaluated ChatGPT’s responses to 100 clinical psychiatric cases, awarding it an “A” rating in 61 cases, with no errors in the diagnoses of different psychiatric disorders and no unacceptable responses, underscoring ChatGPT’s expertise and interpretative capacity in psychiatry. Further supporting this, Schubert et al [118] assessed the performance of ChatGPT 4.0 using neurology board-style examination questions, finding that it answered 85% of the questions correctly, surpassing the average human performance of 73.8%. Meanwhile, in a study of LLMs regarding the prognosis and long-term outcomes of depression, GPT-4, Claude (Anthropic), and Bard (Google AI) showed strong agreement with mental health professionals. They all recommended a combination of psychotherapy and antidepressant medication in every case [130]. This not only proves the reliability of LLMs for mental health assessment but also highlights their usefulness in providing valuable support and guidance for individuals seeking information or coping with mental illness.

However, the direct deployment of LLMs, such as ChatGPT, in clinical settings carries inherent risks. The outputs of LLMs are heavily influenced by prompt engineering, which can lead to inconsistencies that undermine clinical reliability [102,105-107,109]. For example, Farhat et al [105] conducted a critical evaluation of ChatGPT’s ability to generate medication guidelines through detailed cross-questioning and noted that altering prompts substantially changed the responses. While ChatGPT typically provided helpful advice and recommended seeking expert consultation, it occasionally produced inappropriate medication suggestions. Perlis et al [129] verified this, showing that GPT-4 Turbo suggested medications that were considered less efficient choices or contraindicated by experts in 12% of the cases. Moreover, LLMs often lack the necessary clinical judgment capabilities. This issue was highlighted in the study by Grabb [109], which revealed that despite built-in safeguards, ChatGPT remains susceptible to generating extreme and potentially hazardous recommendations. A particularly alarming example was ChatGPT advising a patient with depression to engage in high-risk activities such as bungee jumping as a means of seeking pleasure [109]. These LLMs depend on prompt engineering [102,105,109], which means their responses can vary widely depending on the wording and context of the prompts given. The system prompts, which are predefined instructions given to the model, and the prompts used by the experimental team, such as those in the study by Farhat et al [105], guide the behavior of ChatGPT and similar LLMs. These prompts are designed to accommodate a variety of user requests within legal and ethical boundaries. However, while these boundaries are intended to ensure safe and appropriate responses, they often fail to align with the nuanced sensitivities required in psychological contexts. This mismatch underscores a significant deficiency in the clinical judgment and control of LLMs within sensitive mental health settings.

Further research into other LLMs in the mental health sector has shown a range of capabilities and limitations. For example, a study by Sezgin et al [111] highlighted Language Model for Dialogue Applications’ (LaMDA’s) proficiency in managing complex inquiries about postpartum depression that require medical insight or nuanced understanding; however, they pointed out its challenges with straightforward, factual questions, such as “What are antidepressants?” [111]. Assessments of LLMs such as LLaMA-7B, ChatGLM-6B, and Alpaca, involving 50 interns specializing in mental illness, received favorable feedback regarding the fluency of these models in a clinical context, with scores >9.5 out of 10. However, the results also indicated that the responses of these LLMs often failed to address mental health issues adequately, demonstrated limited professionalism, and resulted in decreased usability [116]. Similarly, a study on psychiatrists’ perceptions of using LLMs such as Bard and Bing AI (Microsoft Corp) in mental health care revealed mixed feelings. While 40% of physicians indicated that they would use such LLMs to assist in answering clinical questions, some expressed serious concerns about their reliability, confidentiality, and potential to damage the patient-physician relationship [130].

## Discussion

### Principal Findings

**Figure 2:**
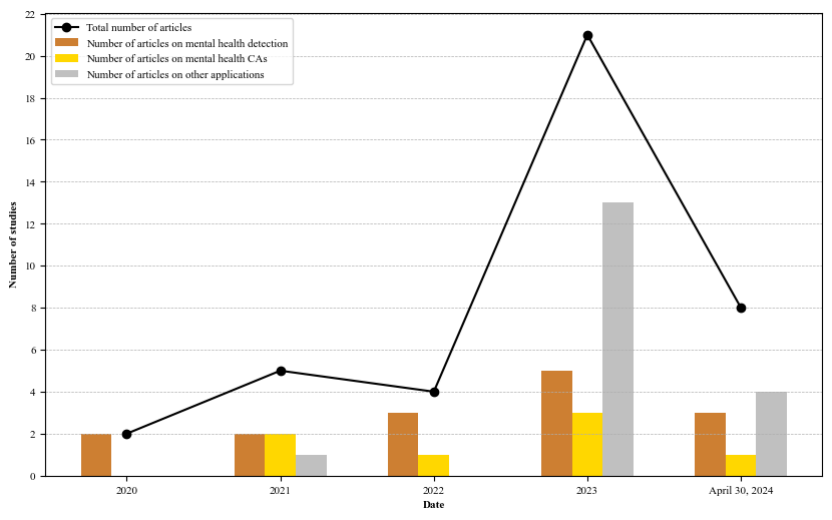
Number of articles included in this literature review, grouped by year of publication and application field. The black line indicates the total number of articles in each year. CA: conversational agent.

In the context of the wider prominence of LLMs in the literature [14,50,57,60,61,69,96,130], this study supports the assertion that interest in LLMs is growing in the field of mental health. Figure 2 indicates an increase in the number of mental health studies using LLMs, with a notable surge observed in 2023 following the introduction of ChatGPT in late 2022. Although we included articles only up to the end of April 2024, it is evident that the number of articles related to LLMs in the field of mental health continues to show a steady increase in 2024. This marks a substantial change in the discourse around LLMs, reflecting their broader acceptance and integration into various aspects of mental health research and practice. The progression from text analysis to a diverse range of applications highlights the academic community’s recognition of the multifaceted uses of LLMs. LLMs are increasingly used for complex psychological assessments, including early screening, diagnosis, and therapeutic interventions.

The findings of this study demonstrate that LLMs are highly effective in analyzing textual data to assess mental states and identify suicidal ideation [50,54,57,60,61,65,66,68,69,72,78], although their categorization often tends to be binary [50,54,65,68,69,72,78]. These LLMs possess extensive knowledge in the field of mental health and are capable of generating empathic responses that closely resemble human interactions [97,98,107]. They show great potential for providing mental health interventions with improved prognoses [50,96,110,127,128,131], with the majority being recognized by psychologists for their appropriateness and accuracy [98,100,129]. The careful and rational application of LLMs can enhance mental health care efficiently and at a lower cost, which is crucial in areas with limited health care capacity. However, there are currently no studies available that provide evaluative evidence to support the clinical use of LLMs.

### Limitations

#### Limitations of Using LLMs in Mental Health

On the basis of the works of literature, the strengths and weaknesses of applying the LLMs in mental health are summarized in Multimedia Appendix 5.

LLMs have a broad range of applications in the mental health field. These models excel in user interaction, provide empathy and anonymity, and help reduce the stigma associated with mental illness [14,107], potentially encouraging more patients to participate in treatment. They also offer a convenient, personalized, and cost-effective way for individuals to access mental health services at any time and from any location, which can be particularly helpful for populations considered socially isolated, especially older adults [60,84,97]. In addition, LLMs can help reduce the burden of care during times of severe health care resource shortages and patient overload, such as during the COVID-19 pandemic [68]. Although previous research has highlighted the potential of LLMs in mental health, it is evident that they are not yet ready for clinical use due to unresolved technical risks and ethical issues.

The use of LLMs in mental health, particularly those fine-tuned for specific tasks such as ChatGPT, reveals clear limitations. The effectiveness of these models heavily depends on the specificity of user-generated prompts. Inappropriate or imprecise prompts can disrupt the conversation’s flow and diminish the model’s effectiveness [75,96,105,107,109]. Even small changes in the content or tone of prompts can sometimes lead to significant variations in responses, which can be particularly problematic in health care settings where interpretability and consistency are critical [14,105,107]. Furthermore, LLMs lack clinical judgment and are not equipped to handle emergencies [95,108]. While they can generally capture extreme emotions and recognize scenarios requiring urgent action, such as suicide ideation [54,65,70,72,78], they often fail to provide direct, practical measures, typically only advising users to seek professional help [96]. In addition, the inherent bias in LLM training data [66,106] can lead to the propagation of stereotypical, discriminatory, or biased viewpoints. This bias can also give rise to hallucinations, that is, LLMs producing erroneous or misleading information [105,131]. Furthermore, hallucinations may stem from overfitting the training data or a lack of context understanding [134]. Such inaccuracies can have serious consequences, such as providing incorrect medical information, reinforcing harmful stereotypes, or failing to recognize and appropriately respond to mental health crises [131]. For example, an LLM might reinforce a harmful belief held by a user, potentially exacerbating their mental health issues. It could also generate nonfactual, overly optimistic, or pessimistic medical advice, delaying appropriate professional intervention. These issues could undermine the integrity and fairness of social psychology [102,105,106,110].

Another critical concern is the “black box” nature of LLMs [105,107,131]. This lack of interpretability complicates the application of LLMs in mental health, where trustworthiness and clarity are important. When we talk about neural networks as black boxes, we know details such as what they were trained with, how they were trained, and what the weights are. However, with many new LLMs, such as GPT-3.5 and 4, researchers and practitioners often access the models via web interfaces or application programming interfaces without complete knowledge of the training data, methods, and model updates. This situation not only presents the traditional challenges associated with neural networks but also has all these additional problems that come from the “hidden” model.

Ethical concern is another significant challenge associated with applying LLMs in mental health. Debates are emerging around issues such as digital personhood, informed consent, the risk of manipulation, and the appropriateness of AI in mimicking human interactions [60,102,105,106,135]. A primary ethical concern is the potential alteration of the traditional therapist-patient relationship. Individuals may struggle to fully grasp the advantages and disadvantages of LLM derivatives, often choosing these options for their lower cost or greater convenience. This shift could lead to an increased reliance on the emotional support provided by AI [14], inadvertently positioning AI as the primary diagnostician and decision maker for mental health issues, thereby undermining trust in conventional health care settings. Moreover, therapists may become overly reliant on LLM-generated answers and use them in clinical decision-making, overlooking the complexities involved in clinical assessment. This reliance could compromise their professional judgment and reduce opportunities for in-depth engagement with patients [17,129,130]. Furthermore, the dehumanization and technocratic nature of mental health care has the potential to depersonalize and dehumanize patients [136], where decisions are more driven by algorithms than by human insight and empathy. This can lead to decisions becoming mechanized, lacking empathy, and detached from ethics [137]. AI systems may fail to recognize or adequately interpret the subtle and often nonverbal cues, such as the tone of voice, facial expressions, and the emotional weightage behind words, which are critical in traditional therapeutic settings [136]. These cues are essential for comprehensively understanding a patient’s condition and providing empathetic care.

In addition, the current roles and accuracy of LLMs in mental health are limited. For instance, while LLMs can categorize a patient’s mood or symptoms, most of these categorizations are binary, such as *depressed* or *not depressed* [50,65]. This oversimplification can lead to misdiagnoses. Data security and user privacy in clinical settings are also of utmost concern [14,54,60,96,130]. Although approximately 70% of psychiatrists believe that managing medical documents will be more efficient using LLMs, many still have concerns about their reliability and privacy [97,130,131]. These concerns could have a devastating impact on patient privacy and undermine the trust between physicians and patients if confidential treatment records stored in LLM databases are compromised. Beyond the technical limitations of AI, the current lack of an industry-benchmarked ethical framework and accountability system hinders the true application of LLMs in clinical practice [131].

#### Limitations of the Selected Articles

Several limitations were identified in the literature review. A significant issue is the age bias present in the social media data used for depression and mental health screening. Social media platforms tend to attract younger demographics, leading to an underrepresentation of older age groups [65]. Furthermore, most studies have focused on social media platforms, such as Twitter, primarily used by English-speaking populations, which may result in a lack of insight into mental health patterns in non–English-speaking regions. Our review included studies in Polish, Chinese, Portuguese, and Malay, all of which highlighted the significant limitations of LLMs caused by the availability and size of databases [54,61,92,98,116]. For instance, due to the absence of a dedicated Polish-language mental health database, a Polish study had to rely on machine-translated English databases [92]. While the LLMs achieve 80% accuracy in categorizing emotions and moods in Polish, this is still lower than the 90% accuracy observed in the original English dataset. This discrepancy highlights that the accuracy of LLMs can be affected by the quality of the database.

Another limitation of this study is the low diversity of LLMs studied. Although we used “large language models” as keywords in our search phase, the vast majority of identified studies (39/40, 98%) focused on BERT and its variants, as well as the GPT model, as one of the models studied. Therefore, this review provides only a limited picture of the variability expected in applicability between different LLMs. In addition, the rapid development of LLM technologies presents a limitation; this study can only reflect current advancements and may not encompass future advances or the full potential of LLMs. For instance, in tests involving psychologically relevant questions and answers, GPT-3.5 achieved an accuracy of 66.8%, while GPT-4.0 reached an accuracy of 85%, compared to the average human score of 73.8% [118]. Evaluating ChatGPT at different stages separately and comparing its performance to that of humans can lead to varied conclusions. In the assessment of prognosis and treatment planning for depression using LLMs, GPT 3.5 demonstrated a distinctly pessimistic prognosis that differed significantly from those of GPT-4, Claude, Bard, and mental health professionals [128]. Therefore, continuous monitoring and evaluation are essential to fully understand and effectively use the advancements in LLM technologies.

### Opportunities and Future Work

Implementing technologies involving LLMs within the health care provision of real patients demands thorough and multifaceted evaluations. It is imperative for both industry and researchers to not let rollout exceed proportional requirements for evidence on safety and efficacy. At the level of the service provider, this includes providing explicit warnings to the public to discourage mistaking LLM functionality for clinical reliability. For example, GPT-4 introduced the ability to process and interpret image inputs within conversational contexts, leading OpenAI to issue an official warning that GPT-4 is not approved for analyzing specialized medical images such as computed tomography scans [138].

A key challenge to address in LLM research is the tendency to produce incoherent text or hallucinations. Future efforts could focus on training LLMs specifically for mental health applications, using datasets with expert labeling to reduce bias and create specialized mental health lexicons [84,102,116]. The creation of specialized datasets could take advantage of the customizable nature of LLMs, fostering the development of models that cater to the distinct needs of varied demographic groups. For instance, unlike models designed for health care professionals that assist in tasks such as data documentation, symptom analysis, medication management, and postoperative care, LLMs intended for patient interaction might be trained with an emphasis on empathy and comfortable dialogue.

Another critical concern is the problem of outdated training data in LLMs. Traditional LLMs, such as GPT-4 (with a cutoff date up to October 2023), rely on potentially outdated training data, limiting their ability to incorporate recent events or information. This can compromise the accuracy and relevance of their responses, leading to the generation of uninformative or incorrect answers, known as “hallucinations” [139]. Retrieval-augmented generation (RAG) technology offers a solution by retrieving facts from external knowledge bases, ensuring that LLMs use the most accurate and up-to-date information [140]. By searching for relevant information from numerous documents, RAG enhances the generation process with the most recent and contextually relevant content [141]. In addition, RAG includes evidence-based information, increasing the reliability and credibility of LLM responses [139].

To further enhance the reliability of LLM content and minimize hallucinations, recent studies suggest adjusting model parameters, such as the “temperature” setting [142-144]. The temperature parameter influences the randomness and predictability of outputs [145]. Lowering the temperature typically results in more deterministic outputs, enhancing coherence and reducing irrelevant content [146]. However, this adjustment can also limit the model’s creativity and adaptability, potentially making it less effective in scenarios requiring diverse or nuanced responses. In mental therapy, where nuanced and sensitive responses are essential, maintaining an optimal balance is crucial. While a lower temperature can ensure accuracy, which is important for tasks such as clinical documentation, it may not suit therapeutic dialogues where personalized engagement is key. Low temperatures can lead to repetitive and impersonal responses, reducing patient engagement and therapeutic effectiveness. To mitigate these risks, regular updates of the model incorporating the latest therapeutic practices and clinical feedback are essential. Such updates could refine the model’s understanding and response mechanisms, ensuring it remains a safe and effective tool for mental health care. Nevertheless, determining the “optimal” temperature setting is challenging, primarily due to the variability in tasks and interaction contexts, which require different levels of creativity and precision.

Data privacy is another important area of concern. Many LLMs, such as ChatGPT and Claude, involve sending data to third-party servers, which poses the risk of data leakage. Current studies have found that LLMs can be enhanced by privacy-enhancing techniques, such as zero-knowledge proofs, differential privacy, and federated learning [147]. In addition, privacy can be preserved by replacing identifying information in textual data with generic tokens. For example, when recording sensitive information (eg, names, addresses, or credit card numbers), using alternatives to mask tokens can help protect user data from unauthorized access [148]. This obfuscation technique ensures that sensitive user information is not stored directly, thereby enhancing data security.

The lack of interpretability in LLM decision-making is another crucial area for future research on health care applications. Future research should examine the models’ architecture, training, and inferential processes for clearer understanding. Detailed documentation of training datasets, sharing of model architectures, and third-party audits would ideally form part of this undertaking. Investigating techniques such as attention mechanisms and modular architectures could illuminate aspects of neural network processing. The implementation of knowledge graphs might help in outlining logical relationships and facts [149]. In addition, another promising approach involves creating a dedicated embedding space during training, guided by an LLM. This space aligns with a causal graph and aids in identifying matches that approximate counterfactuals [146].

Before deploying LLMs in mental health settings, a comprehensive assessment of their reliability, safety, fairness, abuse resistance, interpretability, compliance with social norms, robustness, performance, linguistic accuracy, and cognitive ability is essential. It is also crucial to foster collaborative relationships among mental health professionals, patients, AI researchers, and policy makers. LLMs, for instance, have demonstrated initial competence in providing medication advice; however, their responses can sometimes be inconsistent or include inappropriate suggestions. As such, LLMs require professional oversight and should not be used independently. Nevertheless, when used as decision aids, LLMs have the potential to enhance health care efficiency. This study calls on developers of LLMs to collaborate with authoritative regulators in actively developing ethical guidelines for AI research in health care. These guidelines should aim to adopt a balanced approach that considers the multifaceted nature of LLMs and ensures their responsible integration into medical practice. They are expected to become industry benchmarks, facilitating the future development of LLMs in mental health.

### Conclusions

This review examines the use of LLMs in mental health applications, including text-based screening for mental health conditions, detection of suicidal ideation, CAs, clinical use, and other related applications. Despite the potential of LLMs, challenges such as the production of hallucinatory or harmful information, output inconsistency, and ethical concerns remain. Nevertheless, as technology advances and ethical guidelines improve, LLMs are expected to become increasingly integral and valuable in mental health services, providing alternative solutions to this global health care issue.

## Appendix

Multimedia Appendix 1 PRISMA (Preferred Reporting Items for Systematic Reviews and Meta-Analyses) checklist.

Multimedia Appendix 2 Risk of bias assessment.

Multimedia Appendix 3 Summary of the 40 selected articles from the literature on large language models in mental applications, categorized into each group.

Multimedia Appendix 4 List of the studies excluded at the full-text screening stage.

Multimedia Appendix 5 Summary of the strengths and weaknesses of applying the large language models in mental health.

## Abbreviations

AI: artificial intelligence
BERT: Bidirectional Encoder Representations from Transformers
CA: conversational agent
ERNIE Bot: Enhanced Representation through Knowledge Integration Bot
LaMDA: Language Model for Dialogue Application
LLM: large language model
NLP: natural language processing
PRISMA: Preferred Reporting Items for Systematic Review and Meta-Analyses
RAG: retrieval-augmented generation
ROUGE: Recall-Oriented Understudy for Gisting Evaluation

## Data Availability

The authors ensure that all pertinent data have been incorporated in the manuscript and the multimedia appendices. For access to the research data, interested parties may contact the corresponding author (KL) subject to a reasonable request.
