# Supplementary figures and images for: Large Language Models for Mental Health Applications: Systematic Review
Source: JMIR Ment Health. 2024 Oct 18;11:e57400. doi: 10.2196/57400 (PMC11530718; doi:10.2196/57400)

**Multimedia Appendix 2**

**Supplementary Material 2: Risk of Bias Assessment**


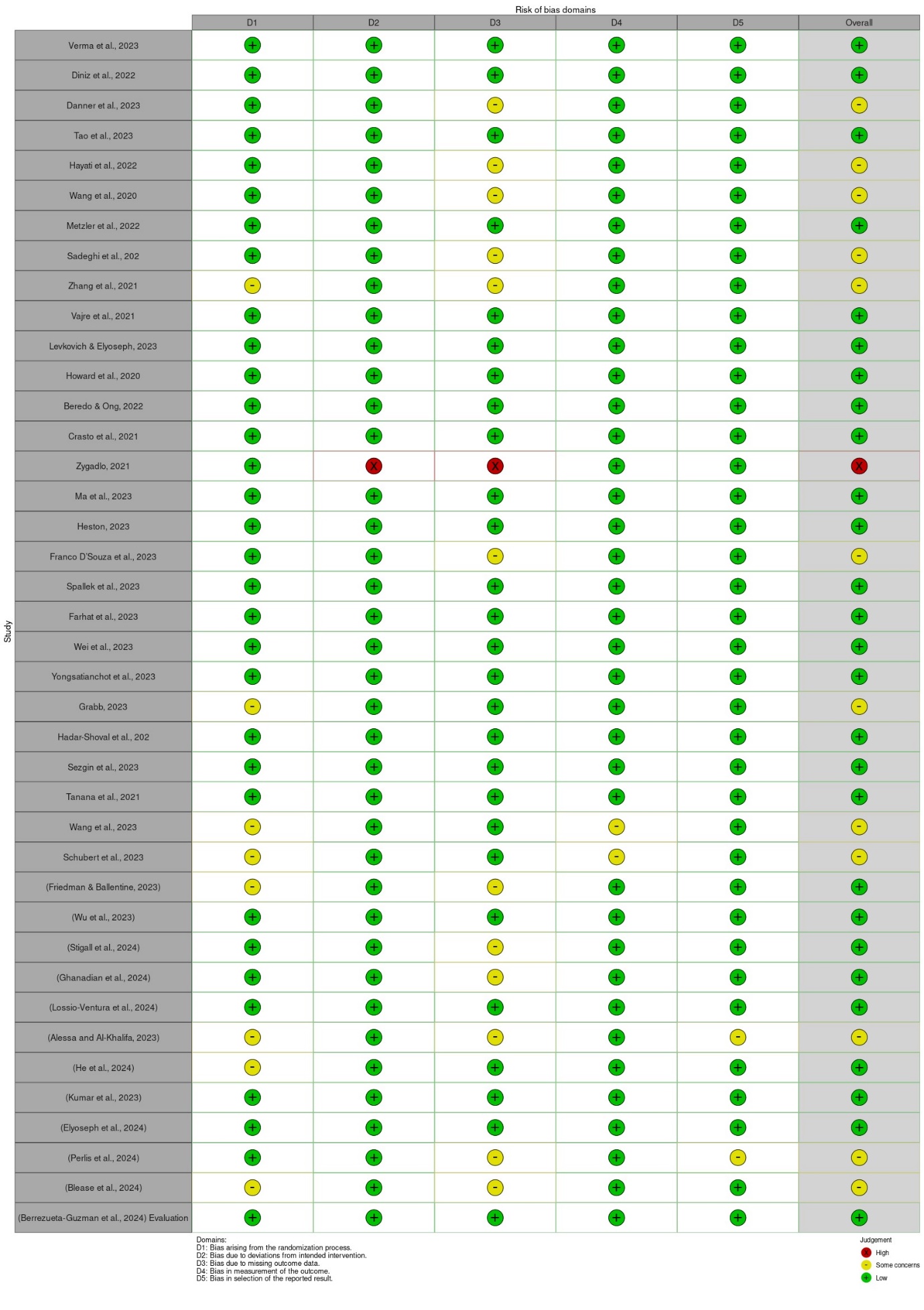

Supplement: Multimedia Appendix 2 [file mental_v11i1e57400_app2.docx]
