# Supplementary material for: Large Language Models for Mental Health Applications: Systematic Review
Source: JMIR Ment Health. 2024 Oct 18;11:e57400. doi: 10.2196/57400 (PMC11530718; doi:10.2196/57400)
Supplement: Multimedia Appendix 3 [file mental_v11i1e57400_app3.docx]

**Multimedia Appendix 3.**

**Supplementary Material 3: Summary of the 40 selected articles from the literature on large language models in mental applications, categorized into each group.**

**Table S1. Summary of the 15 selected articles from the literature on LLMs in mental health conditions and suicidal ideation detection through text.**

| **Ref.** | **Cases** | **Models** | **Data Sources** | **Methodology Used** | **Main Outcomes** |
| --- | --- | --- | --- | --- | --- |
| (Verma et al., 2023) [50] | Detecting depression using LLMs through textual data | RoBERTa [51] | Mental health corpus [52];  Depression Reddit cleaned [53] | This paper used two datasets focused on mental health to train a deep learning model, RoBERTa, for depression detection. Data preprocessing included text cleaning, tokenization, and vectorization, followed by model training with fine-tuning of hyperparameters for binary classification of depression. | The study successfully used a RoBERTa-base model to detect depression with a high accuracy of 96.86%, showcasing the potential of AI in identifying mental health issues through linguistic analysis. |
| (Diniz et al., 2022) [54] | Detecting suicidal ideation using LLMs through Twitter texts | BERT model for Portuguese [55];  Multilingual BERT (base) [29];  BERTimbau [56] | Non-clinical texts from tweets (user posts of the online social network Twitter) | This paper developed the Boamente system through a co-design approach with psychologists, creating a virtual keyboard for text collection and a web platform for data analysis. Texts were collected from Twitter, annotated for suicidal ideation, and processed with LLM techniques. ML and DL models were then trained on this data, allowing mental health professionals to access analysis results without retaining sensitive text data. | The Boamente system demonstrated effective text analysis for suicidal ideation with high privacy standards and actionable insights for mental health professionals. The best-performing BERTimbau Large model (accuracy: 0.955; precision: 0.961; F-score: 0.954; AUC: 0.954) significantly excelled in detecting suicidal tendencies, showcasing robust accuracy and recall in model evaluations. |
| (Danner et al., 2023) [57] | Detecting depression using LLMs through clinical interviews | BERT;  GPT-3.5;  ChatGPT-4 | DAIC-WOZ [58];  Extended-DAIC [59];  simulated data | The method employed deep learning to detect depression symptoms using multimodal datasets. It pre-processed data, addressed class imbalance, fine-tuned the model, and evaluated performance using precision, recall, and F1 score metrics. | The study assessed the abilities of GPT-3.5-turbo and ChatGPT-4 on the DAIC-WOZ dataset, which yielded F1 scores of 0.78 and 0.61 respectively, and a custom BERT model, extended-trained on a larger dataset, which achieved an F1 score of 0.82 on the Extended-DAIC dataset, in recognizing depression in text. |
| (Tao et al., 2023) [60] | Detecting anxiety and depression using LLMs through dialogs in real-life scenarios | ChatGPT | Speech data from nine Q&A tasks related to daily activities (75 patients with anxiety and 64 patients with depression) | The study developed a virtual framework utilizing LLMs to support mental health treatments non-intrusively by analyzing behavioral data and speech features like rate and pitch. This approach, tested with patients from Peking University Sixth Hospital, aimed to detect anxiety and depression symptoms through ChatGPT analysis, facilitating personalized therapeutic strategies. | This paper introduced a virtual interaction framework using LLMs to mitigate negative psychological states. Analysis of Q&A dialogues demonstrated ChatGPT's potential in identifying depression and anxiety. To enhance classification, four language features, including prosodic and speech rate, positively impacted classification. |
| (Hayati et al., 2022) [61] | Detecting depression by Malay dialect speech using LLMs | GPT-3 | Interviews with 53 adults fluent in Kuala Lumpur (KL), Pahang, or Terengganu Malay dialects | Participants were interviewed, their responses transcribed and classified for depression using GPT-3 on a dataset minimally processed to standardize pronouns and remove frequent stop words, with an 80%-20% training-testing split. | GPT-3 was tested on three different dialectal Malay datasets (combined, KL, and non-KL). It performed best on the KL dataset with a max_example value of 10, which achieved the highest overall performance. Despite the promising results, the non-KL dataset showed the lowest performance, suggesting that larger or more homogeneous datasets might be necessary for improved accuracy in depression detection tasks. |
| (Wang et al., 2020) [62] | Detecting depression using LLMs through microblogs | BERT;  RoBERTa [51];  XLNet [63] | 13,993 microblogs collected from the Sina Weibo [64] | The study utilized a dataset from Sina Weibo, annotated for varying levels of depression risk, to evaluate the effectiveness of BERT, RoBERTa, and XLNET models in classifying depression risk through fine-tuning and domain-specific pretraining. | RoBERTa achieved the highest macro-averaged F1 score of 0.424 for depression classification, while BERT scored the highest micro-averaged F1 score of 0.856. Pretraining on an in-domain corpus improved model performance. |
| (Metzler et al., 2022) [65] | Detecting suicidal ideation using LLMs through Twitter texts | BERT;  XLNet [63] | 3202 English tweets | The study involved manually labeling 3,202 English tweets according to a novel scheme, leading to training various machine learning models, including deep learning (BERT, XLNet), for multiclass and binary classification tasks aimed at suicide prevention. | BERT achieved F1-scores of 0.93 for accurately labeling tweets as about suicide and 0.74 for off-topic tweets in the binary classification task. Its performance was similar to or exceeded human performance and matched that of state-of-the-art models on similar tasks. |
| (Sadeghi et al., 2023) [66] | Detecting depression using LLMs through interviews | GPT-3.5-Turbo;  RoBERTa [51] | E-DAIC (219 participants) [67] | The study utilized GPT-3.5-Turbo to transform interview transcripts, making them more informative for detecting depression. It then employed the DepRoBERTa language model, fine-tuned on these transformed transcripts, to predict an individual’s Patient Health Questionnaire (PHQ) score based on text analysis. | The study achieved its lowest error rates, a Mean Absolute Error (MAE) of 3.65 on the dev set and 4.26 on the test set, by fine-tuning DepRoBERTa with a specific prompt, outperforming manual methods and highlighting the potential of automated text analysis for depression detection. |
| (Zhang et al., 2021) [68] | Detecting depression trends using LLMs through Twitter texts | RoBERTa [51];  XLNet [63] | 2575 Twitter users with depression identified via tweets and profiles | The study identified depression-related content on Twitter using regular expressions, built a dataset of 2575 users, trained transformer-based models to classify depression, explored a fusion classifier, and demonstrated the model's ability to monitor depression trends during COVID-19. | This study developed a fusion model that accurately classified depression among Twitter users with 78.9% accuracy. It identified key linguistic and behavioral indicators of depression and demonstrated that depressive users responded to the pandemic later than controls. The findings suggest the model's effectiveness in noninvasively monitoring mental health trends during major events like COVID-19. |
| (Vajre et al., 2021) [69] | Detecting mental health using LLMs through social media texts | PsychBERT | Twitter hashtags and Subreddit (6 domains: anxiety, mental health, suicide, etc) | The paper developed a taxonomy based on HiTOP, implemented a two-stage framework for mental health text identification and behavior detection, and incorporated interpretability components. | The study identified PsychBERT as the highest-performing model, achieving an F1 score of 0.98 in a binary classification task and 0.63 in a more challenging multi-class classification task, indicating its superiority in handling complex mental health-related data. Additionally, PsychBERT's explainability was enhanced by using the Captum library, which confirmed its ability to accurately identify key phrases indicative of mental health issues. |
| (Levkovich & Elyoseph, 2023) [70] | Detecting suicidal ideation using LLMs through text vignette | ChatGPT-3.5;  ChatGPT-4 | ChatGPT's response to the text vignette from Levi-Belz and Gamliel [71] | ChatGPT-4 and ChatGPT-3.5 evaluated a vignette depicting suicide risk, compared to mental health professionals' assessments. | ChatGPT-4's assessments of suicide attempts aligned closely with mental health professionals with an average Z score of 0.01, while ChatGPT-3.5 significantly underestimated these risks with a Z score of -0.83.ChatGPT-4 reported higher rates of suicidal ideation and psychache with Z scores of 0.47 and 1.00, respectively, but assessed resilience levels lower than professionals with Z scores of -0.89 and -0.90. |
| (Howard et al., 2020) [72] | Detecting suicidal ideation using LLMs through social media texts | DeepMoji [73];  Universal Sentence Encoder [74];  GPT-1 | 1588 labeled posts from the Computational Linguistics and Clinical Psychology 2017 shared task | The study utilized sentiment analysis and linguistic tools, along with pre-trained neural network models, to process 1588 posts from a clinical psychology forum. It then used automated machine learning to generate classifiers for efficiently categorizing these posts. | The top-performing system, utilizing features derived from the GPT-1 model fine-tuned on over 150,000 unlabeled Reachout.com posts, achieved a new state-of-the-art macro-averaged F1 score of 0.572 on the CLPsych 2017 task without relying on metadata or preceding posts. However, error analysis indicated that this system frequently misses expressions of hopelessness. |
| (Stigall et al., 2024) [75] | Emotion Classification using LLMs through social media texts | EmoBERTTiny | A collection of publicly available datasets hosted on Kaggle and Huggingface [76, 77] | This paper used a parallel multi-task learning approach with a single loss function for Emotion Classification and Sentiment Analysis. It analyzed the fine-tuned BERTTiny model, EmoBERTTiny, comparing its performance to baseline models and 7B parameter models, benchmarking it against Llama-2-7B-chat and Mistral-7B-Instruct in terms of accuracy, F1-score, precision-recall curves, and inference speed. | EmoBERTTiny outperformed pre-trained and state-of-the-art models in all metrics and computational efficiency, achieving 93.14% accuracy in sentiment analysis and 85.48% in emotion classification. It processes a 256-token context window in 8.04ms post-tokenization and 154.23ms total processing speed. |
| (Ghanadian et al., 2024) [78] | Suicidal ideation detection using LLMs through social media texts | ALBERT; DistilBERT; ChatGPT;  Flan-T5 [79];  Llama | UMD Dataset [80]; Synthetic Datasets (Generated using LLMs like Flan-T5 and Llama2, these datasets augment the UMD dataset to enhance model performance) | This paper detailed a methodology that first extracted social factors from psychology literature to inform GLLM-based data synthesis prompts. It then used three GLLMs to generate synthetic data on suicide-related topics and trained classifiers on real, synthetic, and augmented datasets, testing their performance on both real and synthetic test sets. | The synthetic data-driven method achieved consistent F1-scores of 0.82, comparable to real-world data models yielding F1-scores between 0.75 and 0.87. When 30% of the real-world UMD dataset was combined with the synthetic data, the performance significantly improved, reaching an F1-score of 0.88 on the UMD test set. This result highlights the effectiveness of synthetic data in addressing data scarcity and enhancing model performance. |
| (Lossio-Ventura et al., 2024) [81] | Evaluations of LLMs for sentiment analysis through social media texts | ChatGPT;  Open Pre-Trained Transformers (OPT) | NIH Data Set [82];  Stanford Data Set [83] | This paper created gold standard labels for a subset of each dataset using a panel of human raters. It compared 8 state-of-the-art sentiment analysis tools on both datasets to evaluate variability and disagreement. Additionally, it explored few-shot learning by fine-tuning OPT using a small annotated subset and zero-shot learning using ChatGPT. | This paper revealed high variability and disagreement among sentiment analysis tools when applied to health-related survey data. OPT and ChatGPT demonstrated superior performance, outperforming all other tools. Moreover, ChatGPT outperformed OPT, achieving a 6% higher accuracy and a 4% to 7% higher F-measure. |

**Table S2. Summary of the 7 selected articles from the literature on LLMs in mental health CAs.**

| **Ref.** | **Cases** | **Models** | **Data Sources** | **Methodology Used** | **Main Outcomes** |
| --- | --- | --- | --- | --- | --- |
| (Beredo & Ong, 2022) [84] | Mental health interventions using CAs supported by LLMs | EREN [85];  MHBot [86];  PERMA [87] | Empatheticdialogues (24,850 conversations) [88];  Well-Being Conversations [89];  Perma Lexica [90] | This study evaluated LLMs involves automated evaluation, where metrics like perplexity measure a model's ability to predict unseen test sets, and human evaluation, assessing the chatbot's human-likeness and response quality through criteria like performance, humanity, and affect, evaluated by experts in psychology. Additionally, experts were recruited to assess chatbot interactions based on specific quality attributes, providing a comprehensive understanding of the model's conversational abilities. | This study successfully demonstrated a hybrid conversation model, which combines generative and retrieval approaches to improve language fluency and empathetic response generation in chatbots. This model, tested through both automated metrics and human evaluation, showed that the medium variation of the FTER model outperformed the vanilla DialoGPT in perplexity and that the human-likeness, relevance, and empathetic qualities of responses were significantly enhanced, making VHope a more competent CA with empathetic abilities. |
| (Crasto et al., 2021) [91] | Mental health interventions using CAs supported by LLMs | DialoGPT | Counselchat (includes tags of illness);  question answers from 100 college students | Recognized mental health questionnaires (PHQ-9 & WHO-5) were completed. The DialoGPT fine-tuned with Counselchat data, was employed for chatbot interaction. Micro-interventions were suggested based on identified issues, and a student survey was administered. | The DialoGPT model, demonstrating higher perplexity and preferred by 63% of college participants for its human-like and empathetic responses, was chosen as the most suitable system for addressing student mental health issues. |
| (Zygadlo, 2021) [92] | Mental health interventions using Polish-language CA supported by LLMs | Rasa [93];  spaCy [94];  Transformers;  BERT | EmpatheticDialogues [88];  DailyDialog [95] | The paper entailed developing a chatbot with Rasa and an emotion recognition model, creating a bilingual Polish-English corpus from existing datasets, and employing machine translation for Polish. This approach facilitated sentiment and emotion classification using BERT models, demonstrating the effective use of machine translation for data-scarce languages. | The successful setup of an initial chatbot dialogue framework using Rasa and the development of a bilingual (English and Polish) corpus for emotion recognition. The research has advanced to training BERT-based models for emotion recognition, achieving high accuracy in sentiment and emotion classification, demonstrating the feasibility of integrating machine translation to work with less-resourced languages like Polish for emotional understanding in chatbots. |
| (Ma et al., 2024) [14] | Evaluation of mental health intervention CAs supported by LLMs | GPT-3 | 120 Reddit posts (2913 user comments) | The study utilized a qualitative content analysis of Reddit posts from the r/Replika subreddit to explore user experiences with the AI-based CA Replika, focusing on mental well-being support. By employing a two-stage coding process with a developed codebook, researchers analyzed a representative sample of posts and comments to identify key benefits and challenges. | The study highlighted that CAs like Replika, powered by LLMs, offered crucial mental health support by providing immediate, unbiased assistance and fostering self-discovery. However, they struggled with content filtering, consistency, user dependency, and social stigma, underscoring the importance of cautious use and improvement in mental wellness applications. |
| (Heston, 2023) [96] | Evaluation of mental health intervention CAs supported by LLMs | ChatGPT-3.5 | Public AI mental health CAs from FlowGPT.com | Evaluated ChatGPT-3.5 mental health agents with simulations for recognizing suicidality, tracking referral to humans, and shutdown at risk levels. | This study evaluated 25 Cas from FlowGPT.com, finding that they referred to human intervention at moderate depression levels (PHQ-9 score of 12) and shut down at severe levels (score of 25). Only two agents provided crisis resources, and most resumed dialogue if the risk level decreased. |
| (Alessa and Al-Khalifa, 2023) [97] | Mental health interventions using CAs for the elderly supported by LLMs | ChatGPT; Google Cloud API | Record of interactions with CA; results of the human experts' assessment | This paper explored using ChatGPT to create a chatbot for providing support to older adults and socially isolated seniors. The system incorporated Google's Cloud API for speech recognition and text-to-speech, and personalized prompts based on user information collected through a questionnaire. The chatbot engaged users in empathetic conversations, quizzes, and health tips, with prompt engineering optimized through three experiments. | The proposed ChatGPT-based system effectively serves as a companion for elderly individuals, helping to alleviate loneliness and social isolation. Preliminary evaluations showed that the system could generate relevant responses tailored to elderly personas. |
| **(**He et al., 2024) [98] | Evaluation of CAs handling counseling for people with autism supported by LLMs | ChatGPT | Public available data from the web-based medical consultation platform DXY [99] | This paper selected 100 patient consultation samples related to autism from January 2018 to August 2023. The questions and responses were anonymized and randomized. Three chief physicians assessed the responses across four dimensions: relevance, accuracy, usefulness, and empathy, completing 717 evaluations. The responses were then compared using a Likert scale to gauge their quality. | The study found that 46.86% of assessors preferred responses from physicians, 34.87% favored ChatGPT, and 18.27% favored ERNIE Bot. Physicians and ChatGPT showed higher accuracy and usefulness compared to ERNIE Bot, while ChatGPT outperformed both in empathy. The study concluded that while physicians' responses were generally superior, LLMs like ChatGPT can provide valuable guidance and greater empathy, though further optimization and research are needed for clinical integration. |

**Table S3. Summary of the 18 selected articles from the literature on other applications and evaluation of the LLMs in mental health.**

| **Ref.** | **Cases** | **Model** | **Data Sources** | **Methodology Used** | **Main Outcomes** |
| --- | --- | --- | --- | --- | --- |
| (Franco D’Souza et al., 2023) [100] | Evaluation of ChatGPT's responses to clinical vignettes in psychiatry | ChatGPT 3.5 | 100 Cases in Psychiatry [101];  ChatGPT 3.5 responses to cases | ChatGPT 3.5 responded to 100 psychiatric case vignettes, evaluated by expert faculties across 10 categories using mean scores, and represented graphically. | ChatGPT 3.5 received mostly "Grade A" ratings in 61 out of 100 cases, excelling in management strategies and diagnoses across psychiatric conditions. Few responses received "Grade C" due to minor discrepancies, but no diagnostic errors were noted. |
| (Spallek et al., 2023) [102] | Evaluation of ChatGPT in mental health education | GPT-4 | Real-world data from 'Cracks in the Ice' [103] and 'Positive Choices' [104] | The study utilized GPT-4 and real-world queries and factsheets from two health portals, assessing LLMs’ potential in generating mental health education content within ethical guidelines. | GPT-4's outputs seemed valid but were substandard compared to expert materials, lacking in reading level and adherence to guidelines, requiring careful human editing. Although not suitable for direct consumer queries, GPT-4 can be cautiously used by educators and researchers to develop educational materials, which should disclose AI use and be evaluated for efficacy. |
| (Farhat et al., 2023) [105] | Evaluation of ChatGPT as a complementary mental health resource | ChatGPT | Responses generated by ChatGPT | The study evaluated ChatGPT's effectiveness in mental health support by analyzing its responses and cross-questioning, particularly focusing on issues related to anxiety and depression and its suggestions regarding medications. | ChatGPT displayed significant inconsistencies and low reliability when providing mental health support for anxiety and depression, underlining the necessity of validation by medical professionals and cautious use in mental health contexts. |
| (Wei et al., 2023) [106] | Evaluation of ChatGPT in psychiatry | ChatGPT | Theoretical analysis and literature reviews | The study investigated ChatGPT's application in psychiatry, evaluating its capabilities in screening, diagnosis, and patient support. | The paper found ChatGPT useful in psychiatry, stressing ethical use and human oversight, while noting challenges in accuracy and bias, positioning AI as a supportive tool in care. |
| (Yongsatianchot et al., 2023) [107] | Evaluation of LLMs' perception of emotion | Text-davinci-003 [108];  ChatGPT;  GPT-4 | Responses from three OpenAI LLMs to the Stress and Coping Process Questionnaire | The study assessed the emotional understanding of LLMs like ChatGPT using the Stress and Coping Process Questionnaire (SCPQ) across three OpenAI models (davinci-003, ChatGPT, GPT-4) to compare their appraisal and coping reactions against human data and appraisal theory predictions. | The study applied the SCPQ to three OpenAI LLMs—davinci-003, ChatGPT, and GPT-4—and found that while their responses aligned with human dynamics of appraisal and coping, they did not vary across key appraisal dimensions as predicted and differed significantly in response magnitude. Notably, all models reacted more negatively than humans to negative scenarios, potentially influenced by their training processes. |
| (Grabb, 2023) [109] | Evaluation of prompt engineering by LLMs and its impact on mental health | ChatGPT 4.0 | ChatGPT's answers to 4 unique questions | The study tested ChatGPT 4.0's response variability to four uniquely framed questions about happiness, each asked five times in distinct roles and contexts, to explore the model's adaptability and advice consistency. | The study found ChatGPT 4.0's advice varied widely based on prompt design, emphasizing careful prompt crafting in mental healthcare contexts to ensure safety and relevance. |
| (Hadar-Shoval et al., 2023) [110] | Evaluation of ChatGPT’s mentalizing abilities in borderline personality disorder ( BPD) and schizoid personality disorder (SPD) | ChatGPT 3.5 | Rating of Levels of Emotional Awareness Scale (LEAS) scenarios for BPD and SPD by ChatGPT | The study evaluated ChatGPT's emotional awareness through modified LEAS scenarios for BPD and SPD, scoring responses and analyzing differences in emotion identification and intensity. | ChatGPT was able to accurately describe the emotional reactions of individuals with BPD as more intense, complex, and rich than those with SPD. |
| (Sezgin et al., 2023) [111] | Evaluation of clinical accuracy in LLMs' responses to postpartum depression (PPD) questions | GPT-4 (using ChatGPT);  LaMDA (using Bard) [112] | 14 PPD-related patient-focused frequently asked questions sourced from the American College of Obstetricians and Gynecologists | The study compared responses from GPT-4, LaMDA, and Google Search against ACOG's FAQs on postpartum depression, evaluated by two board-certified physicians using a GRADE-informed scale. Statistical analyses were performed using R software, including interrater reliability and differences in response quality. | ChatGPT outperformed Bard and Google Search in providing high-quality, clinically accurate responses to postpartum depression questions, with significant statistical support and perfect rater agreement on its responses. |
| (Tanana et al., 2021) [113] | Evaluation of LLM’s ability to rate emotions in psychotherapy | BERT;  LIWC [114] | Psychotherapy transcripts that were published by Alexander Street Press [115];  the human ratings from a database of 97,497 utterances from psychotherapy | The paper utilized psychotherapy transcripts to extract utterances for sentiment analysis, employing N-gram models, a recurrent neural network, LIWC, and BERT for comparison. Evaluation metrics included overall accuracy, F1 score, and Cohen's kappa. | MaxEnt models surpassed LIWC, with BERT achieving the highest performance (kappa = 0.48). The best model exceeded human performance on the test set by 14%. |
| (Wang et al., 2020) [116] | Enhancing depression diagnosis and treatment through the use of LLMs | LLaMA-7B;  ChatGLM-6B;  Alpaca;  LLMs+Knowledge | Chinese Incremental Pre-training Dataset [117] | The paper customized a Chinese language model for depression, using datasets and a knowledge graph. Techniques included data augmentation, generating instruction data from the knowledge graph, fine-tuning the model, and reinforcement learning with expert feedback. | The study assessed LLMs' performance in mental health, emphasizing safety, usability, and fluency and integrating mental health knowledge to improve model effectiveness, enabling more tailored dialogues for treatment while ensuring safety and usability. |
| (Schubert et al., 2023) [118] | Evaluation of LLMs' performance on neurology board-style examinations | ChatGPT 3.5;  ChatGPT 4.0 | A question bank from an educational company with 2036 questions that resemble neurology board questions [119] | The study evaluated two LLMs using a neurology question bank, categorizing questions into lower and higher-order types. Statistical analysis compared model performance with human performance. | ChatGPT 4.0 excelled over ChatGPT 3.5, achieving 85.0% accuracy versus 66.8%. It surpassed human performance in specific areas and exhibited high confidence in responses. Longer questions tended to result in more incorrect answers for both models. |
| (Friedman & Ballentine, 2023) [120] | Evaluation of LLMs in data-driven discovery: correlating sentiment changes with psychoactive experiences | BERTowid [29];  BERTiment [121] | Erowid testimonials [122];  drug receptor affinities [123];  brain gene expression data [124];  58K annotated Reddit posts [125] | This paper used BERT and 11,816 testimonials to predict sentiments and demographics, then linked drug effects to words, identifying 11 key factors on a 3D brain model. | This paper found that LLM methods can create unified and robust quantifications of subjective experiences across various psychoactive substances and timescales. The representations learned are evocative and mutually confirmatory, indicating significant potential for LLMs in characterizing psychoactivity. |
| (Wu et al., 2023) [126] | Expanding dataset of Post-Traumatic Stress Disorder (PTSD) using LLMs | GPT- 3.5 Turbo | E-DAIC (219 participants) [67] | This paper developed two text augmentation frameworks utilizing LLMs to address data imbalance in NLP tasks for PTSD diagnosis. The methodologies applied were zero-shot, which generated standardized transcripts, and few-shot, which rephrased existing training samples within the E-DAIC. | This paper demonstrated that two novel text augmentation frameworks using LLMs significantly improved PTSD diagnosis by addressing data imbalances in NLP tasks. The zero-shot approach, which generated new standardized transcripts, achieved the highest performance improvements, while the few-shot approach, which rephrased existing training samples, also surpassed the original dataset's efficacy. |
| (Kumar et al., 2023) 127 | Evaluation of GPT 3 in mental health intervention | GPT 3 | 209 participants responses, with 189 valid responses after filtering | This paper conducted a pilot experiment using a 2x2x2 factorial design to compare LLM-based chatbot interventions with video-based methods for improving mental health awareness. GPT-3 was used to create chatbots for providing mindfulness information and reflection, and participants were recruited from Amazon Mechanical Turk. | This paper found that interaction with either of the chatbots improved participants’ intent to practice mindfulness again, while the tutorial video enhanced their overall experience of the exercise. These findings highlighted the potential promise and outlined directions for exploring the use of LLM-based chatbots for awareness-related interventions. |
| (Elyoseph et al., 2024) [128] | Evaluation of LLMs in mental health intervention | ChatGPT3.5;  ChatGPT4;  Claude;  Bard | ChatGPT 3.5, ChatGPT 4, Claude, Bard, and mental health professionals' responses to text vignettes about depression | This paper conducted a comparative analysis using case vignettes to evaluate the performance of different LLMs against mental health professionals and the general public. The focus was on the LLMs' ability to generate prognoses, anticipated outcomes with and without intervention, and long-term consequences for individuals with depression. | This paper found that ChatGPT-4, Claude, and Bard aligned closely with mental health professionals and the general public in diagnosing depression and recommending combined treatment, while ChatGPT-3.5 had a more pessimistic prognosis. The study highlighted AI's potential to complement mental health professionals but raised concerns about ChatGPT-3.5's impact on patient motivation. |
| (Perlis et al., 2024) [129] | Evaluation of GPT-4 for clinical decision support in bipolar depression | GPT-4 turbo (gpt-4-1106-preview) | Recommendations generated by the augmented GPT-4 model and responses from clinicians treating bipolar disorder | This paper generated 50 vignettes of bipolar disorder cases and had expert clinicians rank treatment options. It then compared these rankings with recommendations from an augmented GPT-4 model using specific guidelines and also evaluated responses from a community clinician group. | This paper found that the augmented GPT-4 model had a Cohen's kappa of 0.31 with expert consensus, identifying the optimal treatment in 50.8% of cases and placing it in the top 3 in 84.4% of cases. In contrast, the base model had a Cohen's kappa of 0.09 and identified the optimal treatment in 23.4% of cases, highlighting the enhanced performance of the augmented model in aligning with expert recommendations. |
| (Blease et al., 2024) [130] | Evaluation of psychiatrists' perceptions of the LLMs | ChatGPT;  Bard;  Bing AI | Survey responses from 138 APA members on LLM chatbot use in psychiatry | This paper surveyed APA members who attended an "AI in Psychiatry" informational session to explore their experiences and opinions on using LLM-powered chatbots in clinical practice. Participants provided feedback through a three-minute survey divided into sections on chatbot usage, its effects on clinical practice, and patient interactions, with responses analyzed using descriptive statistics and thematic analysis. | This paper found that over half of psychiatrists used AI tools like ChatGPT for clinical questions, with nearly 70% agreeing on improved documentation efficiency and almost 90% indicating a need for more training while expressing mixed opinions on patient care impacts and privacy concerns. |
| (Berrezueta-Guzman et al., 2024) [131] | Evaluation of the efficacy of ChatGPT in mental intensive treatment | ChatGPT | Evaluations from 10 attention deficit hyperactivity disorder (ADHD) therapy experts and interactions between therapists and the custom ChatGPT | This paper developed a custom ChatGPT based on a literature review and validated it with therapeutic experts before implementing it in a robotic assistant for ADHD therapies. The Delphi method was used with a panel of ten experts to assess the ChatGPT’s performance across various therapeutic categories, ensuring a thorough, expert-driven evaluation. | This paper found that the custom ChatGPT demonstrated strong capabilities in engaging language use, maintaining interest, promoting active participation, and fostering a positive atmosphere in ADHD therapy sessions, with high ratings in communication and language. However, areas needing improvement were identified, particularly in confidentiality and privacy, cultural and sensory sensitivity, and handling nonverbal cues. |
