# Supplementary material for: Large Language Models for Mental Health Applications: Systematic Review
Source: JMIR Ment Health. 2024 Oct 18;11:e57400. doi: 10.2196/57400 (PMC11530718; doi:10.2196/57400)
Supplement: Multimedia Appendix 4 [file mental_v11i1e57400_app4.docx]

Multimedia Appendix 4

Supplementary Material 4: List of Studies Excluded at the Full-Text Screening Stage

|  | **Title** | | | | | **Reference** | | **Exclusion reason** | | |
| --- | --- | --- | --- | --- | --- | --- | --- | --- | --- | --- |
| 1 | A Novel AI-based chatbot Application for Personalized Medical Diagnosis  and review using Large Language Models | | | | | (S et al., 2023) | | Not  health | about | mental |
| 2 | An Introduction to Generative Artificial Intelligence in Mental Health Care:  Considerations and Guidance | | | | | (King et al., 2023) | | Not only for mental  health | | |
| 3 | Artificial Intelligence Based Analysis of Positive and Negative Tweets  Towards COVID-19 Vaccines | | | | | (Umair & Masciari, 2021) | | Not  health | about | mental |
| 4 | Artificial Intelligence in Psychiatry | | | | | (Briganti, 2023) | | Not about LLMs | | |
| 5 | Assessing the Accuracy of Responses by the Language Model ChatGPT to  Questions Regarding Bariatric Surgery | | | | | (Samaan et al., 2023) | | Not  health | about | mental |
| 6 | ChatGPT and Bard Exhibit  Psychiatry Literature Search | Spontaneous | Citation | Fabrication | during | (McGowan et al., 2023) | | Not  health | about | mental |
| 7 | ChatGPT and mental healthcare: balancing benefits with risks of harms | | | | | (Blease & Torous, 2023) | | It's a review paper,  too short | | |
| 8 | ChatGPT in Answering Queries Related to Lifestyle-Related Diseases and  Disorders | | | | | (Mondal et al., 2023) | | Not  health | about | mental |
| 9 | ChatGPT on ECT: Can Large Language Models Support Psychoeducation? | | | | | (Lundin et al., 2023) | | It's a letter, It's too  short | | |
| 10 | ChatGPT vs Google for Queries Related to Dementia and Other Cognitive Decline: Comparison of Results | | | | | (Hristidis et al., 2023) | | Dementia is not considered a mental  illness | | |
| 11 | ChatGPT vs. Human Annotators: A Comprehensive Analysis of ChatGPT for  Text Annotation | | | | | (Aldeen et al., 2023) | | Not  health | about | mental |
| 12 | Conversational Agents in Health Care: Expert Interviews to Inform the  Definition, Classification, and Conceptual Framework | | | | | (Martinengo et al., 2023) | | Not about LLMs | | |
| 13 | Diagnosing Psychiatric Disorders from History of Present Illness Using a  Large-Scale Linguistic Model | | | | | (Otsuka et al., 2023) | | Not about LLMs | | |
| 14 | Discourse-Level Representations Can Improve Prediction of Degree of  Anxiety | | | | | (Juhng et al., 2023) | | Duplicate | | |
| 15 | Empathy and Equity: Key Considerations for Large Language Model  Adoption in Health Care | | | | | (Koranteng et al., 2023) | | Not  health | about | mental |
| 16 | Ethical Challenges in AI Approaches to Eating Disorders | | | | | (Sharp et al., 2023) | | Not about LLMs | | |
| 17 | Evaluating the Application of Large Language Models in Clinical Research  Contexts | | | | | (Perlis & Fihn, 2023) | | It's a review paper,  too short | | |
| 18 | Examining the Utility of Social Media in COVID-19 Vaccination:  Unsupervised Learning of 672,133 Twitter Posts | | | | | (Liew & Lee, 2021) | | Not about LLMs | | |
| 19 | Global Mental Health Services and the Impact of Artificial Intelligence  Powered Large Language Models | | | | | (van Heerden et al., 2023) | | It's a review paper,  too short | | |
| 20 | Grateful Chatbots: Public Sensemaking through Individual Gratitude  Interventions | | | | | (Schuler &  2023) | Portmann, | Not  health | about | mental |

| 21 | Identifying Rare Circumstances Preceding Female Firearm Suicides: Validating A Large Language Model Approach | (Zhou et al., 2023) | Not about mental health |
| --- | --- | --- | --- |
| 22 | The Impact of Multimodal Large Language Models on Health Care’s Future | (Meskó, 2023) | Not about mental  health |
| 23 | Large Language Models in Medical Education: Opportunities, Challenges,  and Future Directions | (Abd-alrazaq et al., 2023) | Not about mental  health |
| 24 | Linguistic Features of Clients and Counselors for Early Detection of Mental  Health Issues in Online Text-based Counseling | (Shidara et al., 2022) | Not about LLMs |
| 25 | Mental Health Prediction from Social Media Text Using Mixture of Experts | (Santos et al., 2023) | Duplicate |
| 26 | Negatively Correlated Noisy Learners for At-Risk User Detection on Social  Networks: A Study on Depression, Anorexia, Self-Harm, and Suicide | (Ragheb et al., 2023) | Duplicate |
| 27 | Performance of ChatGPT on the Situational Judgement Test—A Professional Dilemmas–Based Examination for Doctors in the United Kingdom | (Borchert et al., 2023) | Not about mental health |
| 28 | Predicting Generalized Anxiety Disorder from Impromptu Speech Transcripts Using Context-Aware Transformer-Based Neural Networks: Model  Evaluation Stud | (Teferra & Rose, 2023) | Not about LLMs |
| 29 | Psychological Insights into The Research and Practice of Embodied  Conversational Agents, Chatbots and Social Assistive Robots: A Systematic Meta-Review | (Kiuchi et al., 2023) | Not about LLMs |
| 30 | Automatic rating of therapist facilitative interpersonal skills in text: A natural  language processing application | (Zech et al., 2022) | Not about mental  health |
| 31 | Social Media Images Can Predict Suicide Risk Using Interpretable Large  Language-Vision Models | (Badian et al., 2023) | Duplicate |
| 32 | Systematic review and meta-analysis of AI-based conversational agents for  promoting mental health and well-being | (Li et al., 2023) | Not about LLMs |
| 33 | Text Dialogue Analysis for Primary Screening of Mild Cognitive Impairment: Development and Validation Study | (C. Wang et al., 2023) | Not about mental health |
| 34 | The Impact of Multimodal Large Language Models on Health Care’s Future | (Meskó, 2023) | Not about mental  health |
| 35 | Transformer-based deep neural network language models for Alzheimer’s disease risk assessment from targeted speech | (Roshanzamir et al., 2021) | Not about mental health |
| 36 | Understanding Dyslexia Through Personalized Large-Scale Computational Models | (Perry et al., 2019) | Not about mental health |
| 37 | Using Generative Artificial Intelligence to Classify Primary Progressive Aphasia from Connected Speech | (Rezaii et al., 2023) | It’s preprint |
| 38 | Waiting for A Digital Therapist: Three Challenges on the Path to  Psychotherapy Delivered by Artificial Intelligence | (Grodniewicz & Hohol,  2023) | Not about LLMs |

| 39 | A Transfer Learning Method for Detecting Alzheimer's Disease Based on  Speech and Natural Language Processing | (Liu et al., 2022) | Not about mental  health |
| --- | --- | --- | --- |
| 40 | Acoustic and Linguistic Analyses to Assess Early-Onset and Genetic  Alzheimer’s Disease | (Pérez-Toro et al., 2021) | Not about mental  health |
| 41 | Using a Chatbot to Provide Formative Feedback: A Longitudinal Study of  Intrinsic Motivation, Cognitive Load, and Learning Performance | (Yin et al., 2024） | Duplicate |
| 42 | Leveraging Large Language Models for Improved Patient Access and Self-  Management: Assessor-Blinded Comparison Between Expert- and AI Generated Content | (Lv et al., 2024) | Not about mental health |
| 43 | Evaluation of Prompts to Simplify Cardiovascular Disease Information  Generated Using a Large Language Model: Cross-Sectional Study | (Mishra et al., 2024) | Not about mental  health |
| 44 | Evaluation of the Performance of Generative AI Large Language Models  ChatGPT, Google Bard, and Microsoft Bing Chat in Supporting Evidence Based Dentistry: Comparative Mixed Methods Study | (Giannakopoulos et al., 2023) | Not about mental health |
| 45 | Beyond Discrimination: Generative AI Applications and Ethical Challenges in  Forensic Psychiatry | (Tortora, 2024) | Not about LLMs |
| 46 | Assessing the Alignment of Large Language Models With Human Values for Mental Health Integration: Cross-Sectional Study Using Schwartz’s Theory of  Basic Values | (Hadar-Shoval et al., 2024) | Not about mental health |
| 47 | Depression and Reciprocal Language Style Matching in Text Messages | (Weinstein and Jensen,  2024) | Not about LLMs |
| 48 | “I Have a Different Perspective as I Am Working Through This” Speech  Language Pathologist Reflections on Autism | (DeThorne et al., 2024) | Not about LLMs |
| 49 | Artificial Intelligence in Medical Education: Comparative Analysis of  ChatGPT, Bing, and Medical Students in Germany | (Roos et al., 2023) | Not about mental  health |
| 50 | An Entity Extraction Pipeline for Medical Text Records Using Large  Language Models: Analytical Study | (Wang et al., 2024) | Not about mental  health |
| 51 | XAI Transformer based Approach for Interpreting Depressed and Suicidal  User Behavior on Online Social Networks | (Malhotra and Jindal,  2024) | Duplicate |
| 52 | University Students’ Acceptance and Usage of Generative AI (ChatGPT) from  a Psycho Technical Perspective | (Faruk et al., 2023) | Not about mental  health |
| 53 | Fairness Evaluation Within Large Language Models through the Lens of  Depression | (Han, 2024) | Too short |
| 54 | A Machine Learning Enabled Approach for Mental and Physical Health  Management Using OpenCV, NLP and IOT | (Rane et al., 2024) | Not about LLMs |
| 55 | Machine Feeling by Knowledge Acquisition with Emotion Map | (Lim et al., 2024) | Not about LLMs |
| 56 | Evaluating Emotional Detection & Classification Capabilities of GPT-2 &  GPT-Neo Using Textual Data | (Jain et al., 2024) | Duplicate |
| 57 | Development of Serious Game Theory Framework in Virtual Reality for  Alzheimer's Patients | (Zuo et al., 2024) | Not about LLMs |
| 58 | Calibration of Transformer-Based Models for Identifying Stress and  Depression in Social Media | (Ilias et al., 2023) | Duplicate |
| 59 | ALTRUIST: a Python package to emulate a Virtual Digital Cohort Study using  social media data | (Bour et al., 2024) | Not about LLMs |

| 60 | Large Language Models and Healthcare Alliance: Potential and Challenges of  Two Representative Use Cases | (García-Méndez and de  Arriba-Pérez, 2024) | Not about mental  health |
| --- | --- | --- | --- |
| 61 | A Platform for Connecting Social Media Data to Domain-Specific Topics  Using Large Language Models: An Application to Student Mental Health | (Ruocco et al., 2024) | Duplicate |

LLMs=large language models
