# Supplementary material for: Large Language Models for Mental Health Applications: Systematic Review
Source: JMIR Ment Health. 2024 Oct 18;11:e57400. doi: 10.2196/57400 (PMC11530718; doi:10.2196/57400)
Supplement: Multimedia Appendix 5 [file mental_v11i1e57400_app5.docx]

Multimedia Appendix 5

Supplementary Material 5: Summary of main strengths, limitations, and suggestions of LLMs in mental health from the selected articles.

| Category | strength | limitation | suggestion |
| --- | --- | --- | --- |
| Mental Illness and Suicidal Ideation Detection | - High Detection Accuracy: Advanced LLMs achieve high accuracy in detecting depression and other mental health issues from textual data [50,57]. - Early Detection Capabilities: LLMs effectively enable early detection of suicidal ideation and depression, crucial for timely interventions [54,66,78]. - Multilingual and Diverse Data Handling: Demonstrated capability of LLMs in handling data across different languages and cultural contexts, improving global mental health monitoring [60,61]. | - Privacy and Ethical Concerns: Ethical concerns due to the passive collection of sensitive text data, underscore the need for improved privacy controls [54,60]. - Contextual and Emotional Understanding Deficits: Limitations in missing deeper contextual and emotional nuances critical for accurate mental health assessments [60,65,75]. - Generalizability Challenges: Challenges in generalizing across diverse populations due to training on specific datasets [65,67,78,81]. | - Improve Privacy and Ethics: Focus on enhancing privacy controls and adhering to ethical standards in future developments [54,60]. - Enhance Contextual Understanding: Incorporate multimodal data to improve models' understanding of context and emotions [60,65]. - Expand Cross-cultural and Multilingual Research: Advance models with diverse datasets to improve universality and adaptability [60,61,78]. |
| Mental Health CAs | - Enhanced Empathy and Engagement: LLMs like VHope and Replika offer empathetic and human-like interactions [14,84,98]. - Accessibility and Reach: LLMs can provide support at scale and across different languages, making mental health support more accessible to diverse populations [92,97]. - Complex Inquiry Handling: LLMs are adept at handling complex medical and emotional inquiries, providing nuanced responses based on extensive datasets [84,98]. | - Inconsistency and Reliability: LLMs like ChatGPT can produce inconsistent and sometimes unreliable outputs, especially in high-stakes scenarios such as diagnosing or managing mental health conditions [96]. - Lack of Deep Understanding: LLMs struggle with understanding context deeply and can give inappropriate responses, lacking the sensitivity required for certain mental health interactions [14]. - Inadequate Crisis Response and Safety Protocols: LLMs may not adequately identify or respond appropriately to severe mental health crises, which can be dangerous if the system fails to refer users to human intervention in a timely and effective manner [96]. | - Enhanced Model Testing and Validation: Implement comprehensive testing protocols and simulations of mental health scenarios to improve the consistency and reliability of LLM outputs [96,97]. - Advanced Contextual Understanding: Develop and integrate advanced algorithms for LLMs to enhance their ability to comprehend and respond appropriately to the complex nuances of mental health conversations [92,98]. - Robust Crisis Management Integration: Integrate sophisticated algorithms for crisis detection and escalation within LLMs, working in partnership with healthcare professionals to ensure these systems align with clinical standards [96]. |
| Other Applications of LLMs in Mental Health | - Diverse Applications: LLMs have shown broad applications in mental health, excelling in diagnostic aid, therapeutic strategy development, and educational material creation, which could enhance both patient care and medical training. [100,102,106,111,118,127,128,129,130,131]. - Emotional and Behavioral Insights: Some LLMs effectively mimic and understand human emotional and behavioral patterns, supporting their use in psychotherapy to analyze and respond to patient emotions accurately [107,113,128]. - Enhance the Quality and Diversity of Datasets: LLMs can generate synthetic clinical data through advanced text enhancement techniques and knowledge-enhanced training, reducing costs and increasing data availability [116,126]. | - Generalizability and Bias Concerns: The specific dataset or context used and inherent biases in the training data may affect the fairness and accuracy of LLM outputs [109,110,116,127,128,131]. - Consistency and Reliability Issues: The outputs of LLMs might vary when generating diagnostic recommendations or treatment strategies, requiring thorough validation and supervision [105,109]. - Ethical and Safety Risks: The deployment of LLMs in mental health contexts introduces significant concerns, including the risk of violating data privacy, the potential for generating harmful advice, and the challenges of adhering to ethical standards [102,106,131]. | - Expanding Research Scope and Diversity: Increasing application research in different datasets, different populations, and clinical settings to ensure the generalizability and applicability of LLMs [102,107,127,128,129,131]. - Human Oversight and Annotation: Creating specialized, clinically relevant training datasets with expert input and oversight to ensure accurate and stable model outputs [102,105,106,109,129]. - Developing Integration and Regulatory Frameworks: Developing detailed guidelines and frameworks to ensure the ethical and safe use of AI in clinical practices and to complement rather than replace human healthcare providers [106,111,131]. |
